# Therapeutic Potential of Vitamin D in Management of Asthma: A Literature Review

**DOI:** 10.7759/cureus.41956

**Published:** 2023-07-16

**Authors:** Charu Tibrewal, Naisargi Shrikant Modi, Parth S Bajoria, Prathma Anandbhai Dave, Ralph Kingsford Rohit, Priyansh Patel, Siddharth Kamal Gandhi, Sai Dheeraj Gutlapalli, Peter Gottlieb, Jay Nfonoyim

**Affiliations:** 1 Department of Internal Medicine, Civil Hospital Ahmedabad, Ahmedabad, IND; 2 Department of Internal Medicine, GMERS Medical College Gandhinagar, Gandhinagar, IND; 3 Department of Internal Medicine, Medical College Baroda, Vadodara, IND; 4 Department of Internal Medicine, Dayanand Medical College and Hospital, Ludhiana, IND; 5 Department of Internal Medicine, California Institute of Behavioral Neurosciences & Psychology, Fairfield, USA; 6 Department of Internal Medicine, Shri M.P. Shah Government Medical College, Jamnagar, IND; 7 Department of Internal Medicine, Richmond University Medical Center, Staten Island, USA; 8 Department of Pulmonary and Critical Care Medicine, Richmond University Medical Center, Staten Island, USA

**Keywords:** vitamin d, prevention, therapeutics, acute asthma managment, asthma

## Abstract

Asthma, a prevalent chronic respiratory illness, affects a substantial number of individuals worldwide, with an estimated occurrence of 358 million cases. Evidence for the benefits of vitamin D in treating asthma is ambiguous and contradictory. The goal of this review article is to emphasize the value of vitamin D supplementation for people with asthma. Medical subject headings (MeSH) terminology was used to search the PubMed Central, MEDLINE, and PubMed databases for articles on vitamin D supplementation in asthma patients. We selected a comprehensive range of academic writing examples published in English, encompassing various types of texts. The study included a total of 37 papers, of which 18 were randomized controlled trials (RCTs) and five were meta-analyses. The use of a corticosteroid, with or without the inclusion of an adrenergic receptor agonist, improves the disease's symptoms, but it is unable to halt the long-term decline in lung function. Over the past 20 years, vitamin D has developed into a potent immunomodulator, influencing both immunological and structural cells, most notably airway smooth muscle cells. Among adults with low 25-hydroxyvitamin D levels, the administration of vitamin D supplements was found to have positive effects in a reduction in the likelihood of asthma exacerbations requiring systemic corticosteroids. The provision of vitamin D supplements during pregnancy significantly reduces the risk of asthma in babies. Both children and adults with inadequate vitamin D levels who have been given vitamin D supplements have shown evident preventive effects against asthma. Therefore, we conclude there should be a lower threshold for prescribing vitamin D to patients with asthma who are vitamin D deficient.

## Introduction and background

With the discovery of vitamin D in 1920, it was originally classified as a vitamin because it is essential for maintaining calcium homeostasis and normal bone development [1]. It has since been discovered that vitamin D is not technically a vitamin, as exposure to UV rays can cause the body to synthesize it. In fact, vitamin D is a steroid, namely, a secosteroid [1]. The list of novel biological effects of vitamin D is constantly expanding and includes nephroprotection; improvement of glucose metabolism; endothelial and cardiovascular protection; immunomodulatory effects on lymphocytes, macrophages, and dendritic cells; promotion of *Mycobacterium tuberculosis* killing by cathelicidin; and antipruritic properties [2]. Vitamin D also traditionally facilitates the absorption of minerals, especially calcium, iron, magnesium, and zinc, from the gastrointestinal tract. The perception of it being a panacea is unsurprising [2]. Asthma is a widespread and versatile non-infectious disease. Variable airway symptoms, such as cough, wheezing, chest tightness, and decreased airflow, are hallmarks of asthma and result from persistent airway inflammation, tissue remodeling, bronchial hyperresponsiveness, and excessive mucus secretion [3]. Asthma was the most prevalent chronic respiratory condition, with an estimated prevalence of 25 million cases, according to the Centers for Disease Control and Prevention (CDC) national asthma data 2021 [4].

A growing body of research on epidemiologic associations between vitamin D deficiency and poor asthma control supports the therapeutic efficacy of vitamin D in the treatment of asthma [5]. Vitamin D regulates the function of lymphocytes, mast cells, antigen-presenting cells, and structural cells in heterogeneous asthma endotypes to control excessive inflammatory responses, providing evidence for a causal relationship [5]. Exacerbations, also known as periods of sudden worsening of symptoms, are a leading cause of death in asthma and are often caused by viral infections of the upper respiratory tract. Interleukin-17A (IL-17A), a proinflammatory cytokine that exacerbates allergic airway responses, is produced more frequently during viral asthma exacerbations [6]. When isolated peripheral blood mononuclear cells from severe asthma patients are exposed to vitamin D metabolites, these cells do not produce IL-17A. Instead, vitamin D metabolites support antiviral responses in airway epithelial cells [6]. These mechanisms provide evidence for the involvement of vitamin D as a secondary preventive agent in mitigating asthma aggravations and inflammation [5]. Some studies have shown that vitamin D treatment reduces asthma exacerbations by 30% in patients with mild to moderate asthma and low vitamin D levels [7]. However, research results are conflicting and do not conclusively prove that vitamin D is helpful in treating asthma [8]. Clinical trials involving children, pregnant women, and adults have demonstrated limited or negligible enhancements in asthma symptoms, disease onset, or progression following vitamin D administration [8]. Despite observational studies consistently finding significant associations between low vitamin D levels and increased susceptibility to asthma, randomized clinical trials examining the effects of vitamin D supplementation on the risk of asthma have produced contradictory results. The purpose of our review article is to highlight the importance of vitamin D supplementation among asthmatics.

Methodology

In conjunction with all authors, PubMed Central, MEDLINE, and PubMed databases were searched in May 2023 using various combinations of vitamin D and asthma. A total of 1,476 papers were identified, and a free full-text filter was applied to yield 809 papers. However, the following search strategy was selected based on the medical subject headings (MeSH) vocabulary: ("Vitamin D/immunology" [Majr] OR "Vitamin D/therapeutic use" [Majr]) AND ("Asthma/drug therapy" [Majr] OR "Asthma/prevention and control" [Majr] OR "Asthma/therapy" [Majr]). No time limits were set. We selected all types of study literature that was published in English with full text. Articles were excluded where the free full text could not be retrieved. Duplicate publications and gray literature were also excluded. All the articles underwent screening, and any disagreements among the authors were resolved through discussion until a consensus was reached. After discussion among all the authors, a total of 37 studies were unanimously included in the review. Of those 37 studies, 18 were randomized controlled trials (RCTs), five were meta-analyses, and four were systemic reviews.

## Review

As a chronic inflammatory illness of the airways, bronchial asthma involves a large number of inflammatory cells. The primary cause of asthma is an unbalanced T-helper (Th1/Th2) ratio, which causes the production of Th2 cytokines such as IL-4 and IL-13 while inhibiting the production of Th1 cytokines such as interferon (IFN) [9]. In the last few years, asthma incidence has significantly increased, making it one of the most serious and prevalent respiratory disorders. Research on the prevention and treatment of asthma is becoming more and more vital since it has a significant and growing negative economic and social impact [9].

Disease burden and pathophysiology

With yearly emergency department (ED) visit rates of 23-42 per 1000, preschool-aged children experience the greatest rates of ED visits and hospitalizations due to wheezing or asthma among all age groups; typically, one in three hospitalizations follow an ED visit [10]. This is not surprising given that wheezing, the most prevalent asthma symptom among preschoolers, affects up to 50% of children before the age of six [11]. Children's viral upper respiratory tract infections (URTIs) cause more than 80% of asthma attacks [11]. Preschoolers with recurrent wheezing or asthma can experience up to eight URTIs annually, with rhinovirus being the most prevalent cause. Investigating preventive measures is thus necessary to lessen the frequency and severity of virally generated asthma exacerbations in preschool-aged children [11]. For a considerable period of time, it has been widely recognized that airway smooth muscle cells (ASM) play a central role in contracting in response to both local and circulating triggers. ASM cells are crucial to the integrity and shape of the airways. These factors regulate broncho-motor tone and play a role in the distinctive hyperresponsiveness observed in the airways of individuals with asthma [12]. ASM cells and innate and adaptive immune response cells communicate with one another to some extent. It has been demonstrated that mediators (IL-6 and IL-8) generated by ASM cells activate and draw mast cells and leukocytes to the airways, causing airway inflammation and hyperresponsiveness. This creates the conditions for later chronic inflammation and airway remodeling, which are characterized by structural modifications in the airways that lead to ASM cell hypertrophy and hyperplasia [12].

Vitamin D physiology

Vitamin D precursor can be obtained from two primary origins: Intake through diet from sources such as fish oils, fish liver, egg yolk, and dietary supplements and through the skin's exposure to UVB radiation [8]. Following exposure to UVB radiation, 7-dehydrocholesterol undergoes conversion in the skin, giving rise to pre-vitamin D3, initiating the process of vitamin D synthesis. The pre-vitamin D3, referred to as cholecalciferol, undergoes a thermally induced isomerization process to transform into vitamin D3. The liver subsequently undergoes hydroxylation to convert vitamin D3 to 25-hydroxyvitamin D, which is then released into the bloodstream. 25-Hydroxyvitamin D must attach to the vitamin D-binding protein (VDBP) to maintain stability in the circulation [8]. However, between November and March, the skin produces little vitamin D, especially in the north. Therefore, vitamin D levels drop significantly during the winter in temperate locations where exposure to sunshine is limited for a considerable portion of the year. As a result, dietary sources of vitamin D become crucial for preventing insufficiency [13]. The enzyme 1-hydroxylase performs a second hydroxylation largely in the kidney to create 1,25-dihydroxyvitamin D3 also known as calcitriol, the active metabolite. Calcitriol interacts with VDR in the cytoplasm to display its biological activity intracellularly [8]. This intricate structure combines with the retinoid X receptor, which subsequently translocates to the nucleus. Inside the nucleus, it has the ability to attach to vitamin D response elements, thereby regulating the transcription of the target gene either by activation or repression [8]. While calcitriol is the active form, it does not serve as a representation of vitamin D stores. Vitamin D serum levels are frequently determined by measuring circulating levels of 25-hydroxyvitamin D, which indicate vitamin D reserves [13].

Vitamin D levels and deficiency

Vitamin D deficiency is defined as a blood vitamin D concentration below 20 ng/mL (less than 50 nmol/L), whereas vitamin D insufficiency is defined as a serum vitamin D concentration over 20 but below 30 ng/mL [14]. Despite the fact that recent research has revealed more important details about vitamin D and its mode of action, both insufficiency and deficiency of vitamin D have become more prevalent in the world's population [14]. According to several studies, even in sunny places, vitamin D insufficiency is still common in developing nations, despite multivitamin use and food augmentation. It might be connected to alterations in lifestyle, such as less exposure to sunlight, more time spent indoors, dietary changes, and the usage of sunscreen [14]. However, due to ambiguities in techniques for measuring total vitamin D concentrations, the use of blood vitamin D3 concentrations as a biomarker of vitamin D status has come under scrutiny [15]. In contrast, the free hormone hypothesis states that only free quantities of vitamin D3 will diffuse across cell membranes and proceed through metabolism to become calcitriol, which will then activate the vitamin D receptor. So, as opposed to total serum concentrations, free vitamin D3 concentrations are thought to be more accurate indicators of vitamin D status [15]. It is preferable to avoid deficiencies or insufficiencies than to necessitate treatment. Considering that only a limited number of foods contain significant quantities of vitamin D, sunlight is the optimal natural source of the vitamin for humans. When exposed to enough sunshine to cause mild erythema on a light-skinned person wearing little clothes (such as a bathing suit), the body creates endogenous vitamin D that is equivalent to consuming 10,000-25,000 IU [16]. Despite equivalent exposure, individuals with darker skin produce significantly lower amounts of vitamin D. In the United States, the fortification of milk with vitamin D began in the 1930s in response to the prevalence of rickets as a major concern. In higher latitudes, further wheat fortification has been advocated as a cost-effective method of preventing vitamin D insufficiency [16]. For children identified as deficient in vitamin D, the Endocrine Society suggests a regimen of weekly boluses of 50,000 IU of vitamin D2 for a duration of six weeks, followed by daily supplements of 1,000 IU of vitamin D3. However, this regimen is probably insufficient to raise serum vitamin D levels to anti-inflammatory levels [16].

Current treatment options for asthma

Treating the symptoms of asthma involves the administration of a corticosteroid, commonly used to manage asthma symptoms, either alone or in combination with an adrenergic receptor agonist [8]. The adrenergic receptor agonist can be a short-acting type, which alleviates bronchoconstriction, or a long-acting type, which offers sustained control over contractions. Despite the fact that these medications offer a wide spectrum of anti-inflammatory characteristics, they cannot stop the long-term deterioration of lung function [8]. By far, the most commonly used medication to treat asthma is corticosteroids. Patients with moderate-to-severe asthma may benefit from further therapy with anticholinergic drugs such as tiotropium [12]. The safety and efficacy of using these medications in children and older individuals remain uncertain. Vitamin D has become a powerful immunomodulator during the past 20 years, controlling both immunological and structural cells, particularly ASM cells [12]. Better treatment alternatives are required to tackle the disease, as asthma incidence is rising globally.

Role of vitamin D in asthma and immunomodulation

**Figure 1:**
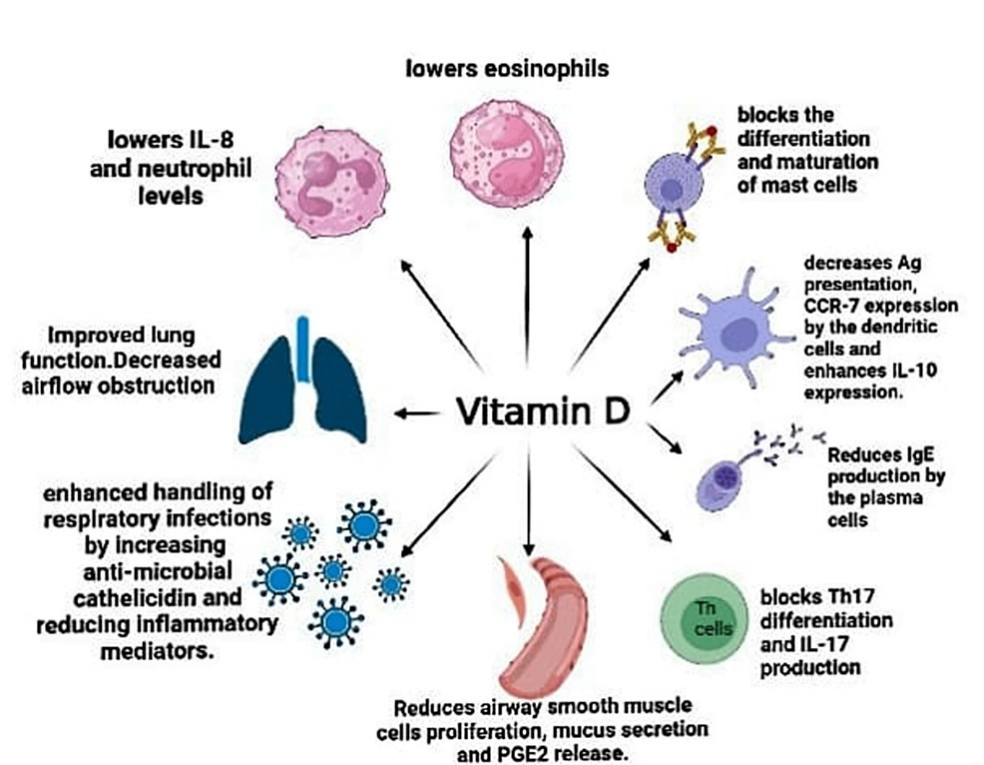
Immune-regulating activity of vitamin D on inflammatory cells involved in the pathogenesis of asthma. IL: interleukin, Ag: antigen, PGE2: prostaglandin E2, Th17: T helper cell 17, IgE: immunoglobulin E, CCR7: C-C chemokine receptor 7 Image credits: Charu Tibrewal, Priyansh Patel, Naisargi Shrikant Modi

In addition to maintaining bone and calcium levels, vitamin D also regulates inflammation and innate and adaptive immunological responses. One of the ways vitamin D works is through its metabolite calcitriol, which binds to the vitamin D receptor and functions as a transcription factor, activating genes that are responsive to vitamin D and are found in the majority, if not all, immune system cells [17]. Additionally, calcitriol controls T-lymphocyte activity and proliferation while reducing inflammation and cytokine expression. These actions govern the adaptive immune system. Vitamin D, the parent molecule, is a powerful and widespread modulator of endothelium stability and barrier function, in addition to the roles it plays as calcitriol [17]. In children with asthma, allergen-specific immunoglobulin E (IgE) and total IgE are important indicators. A crucial therapeutic objective in children with severe asthma is total IgE too. The likelihood of severe asthma exacerbations in children, particularly those brought on by viral infections, is decreased by omalizumab, a monoclonal antibody that binds to circulating IgE [18]. Vitamin D has been demonstrated in studies to stimulate regulatory T-cells (Treg), lessen Th2 and Th17 immunological responses, and enhance the production of IL-10, all of which could lower IgE levels [18]. Furthermore, it has been demonstrated that allergic airway illness is associated with a heightened vulnerability to infections. This may be partially explained by the finding that allergic inflammation reduces the local host's ability to fight off infections by lowering the expression of antimicrobial peptides and proteins (AMPs), which is at least in part mediated by Th2 cytokines [19]. These AMPs are a crucial component of innate immunity in most multicellular creatures, are mostly produced by neutrophils and epithelial cells, and are effective against a variety of bacteria, fungi, viruses, and microorganisms. The production of AMPs such as neutrophil gelatinase-associated lipocalin (LCN2) and cathelicidin (hCAP18/LL-37) in epithelial cells has been shown to be significantly regulated by vitamin D [19]. The process of lung development initiates during fetal development and continues throughout the early years of life. Mammalian lung development progresses through five distinct anatomical phases: embryonic (four-seven weeks), pseudoglandular (7-17 weeks), canalicular (17-26 weeks), saccular (27-36 weeks), and alveolar (36 weeks to approximately two years) [20]. The alveolar epithelium undergoes rapid differentiation at the conclusion of fetal lung development as part of the setup for postpartum gas exchange. Type II pneumocyte differentiation, together with the beginning of surfactant synthesis and the increasing removal of glycogen, are all aspects of fetal pulmonary maturation [20]. Previous research findings are strongly reinforced by animal studies, which further demonstrate the link between vitamin D deficiency and respiratory distress in preterm infants (rachitic respiratory distress). These studies also indicate that vitamin D plays a crucial role in the development of the lungs during the late stages of pregnancy, specifically the saccular and alveolar phases of lung development [20]. Another study showed that vitamin D reduced airway inflammation in asthmatic mice by enhancing the Th17/Treg balance and suppressing the NF-B pathway [21]. Studies have demonstrated that vitamin D has the ability to impede the progression of the cell cycle in ASM cells, thereby diminishing ASM cell proliferation. It has also been shown to reduce the production of powerful inflammatory mediators such as tumor necrosis factor (TNF), IL-8, and RANTES (regulated upon activation, normal T-cell expressed and secreted), which reduces the chemotaxis of inflammatory cells to the airways [12]. Vitamin D has been found to regulate the expression of factors involved in collagen accumulation and airway remodeling. This regulatory effect leads to a reduction in asthma-related airway remodeling, airway hyperresponsiveness, and overall inflammation [12]. The studies suggest that antenatal vitamin D supplementation may potentially reduce the risk of early childhood asthma or wheezing by influencing sphingolipid metabolism, as indicated by the 17q21 genotype. The proposed mechanism underlying this effect is the alteration of sphingolipid metabolism [22]. Sphingolipid formation depends on a critical process that is catalyzed by serine palmitoyltransferase. Serine palmitoyltransferase activity is inhibited by ORMDL3, which results in a reduction in the generation of sphingolipids via the de novo pathway. Through the sphingolipid metabolism route, prenatal vitamin D supplementation enhances the formation of sphingolipids in offspring, decreasing the risk of asthma [22]. When ORMDL3 is upregulated, it leads to the suppression of the sphingolipid metabolism pathway, thereby preventing an increase in sphingolipid synthesis. Consequently, vitamin D does not provide a shielding effect against the risk of asthma [22]. Figure 1 emphasizes the immune-regulating effects of vitamin D on inflammatory cells associated with asthma [16,23].

Analysis

Vitamin D has been identified as a powerful regulator of the inflammatory response found in allergic airway inflammation based on the results of numerous in vitro and in vivo studies. However, Forno et al. found that, in children with chronic asthma and low vitamin D levels, vitamin D3 treatment did not significantly prolong the time to a severe asthma exacerbation when compared to a placebo. The outcomes do not support the use of vitamin D3 therapy to stop severe asthma exacerbations in this patient population [24]. According to Rosser et al., vitamin D treatment (4,000 IU/day) showed no appreciable effects on total IgE in kids with persistent asthma and low vitamin D levels when compared to placebo [18]. According to a systematic review and meta-analysis conducted by Fares et al., the current evidence does not support the routine use of vitamin D supplementation in children with asthma. In all instances, doctors should think about giving vitamin D supplements to kids who have low amounts of the vitamin [25]. The World Allergy Organization affirms that there is a lack of evidence to substantiate the notion that vitamin D supplementation can reduce the likelihood of allergic disease development in children [14]. Additionally, it has been claimed that vitamin D supplementation in expectant moms, healthy infants, and nursing mothers has not protected against allergic disorders [14]. The VIDA randomized clinical trial conducted by Castro et al. found that administering vitamin D3 to adults with persistent asthma and low vitamin D levels did not reduce the risk of initial treatment failure or exacerbation. These results contradict a therapeutic vitamin D3 supplementation strategy for asthmatic patients [26]. Jolliffe et al. discovered, however, that vitamin D administration reduced the likelihood of asthma exacerbations requiring systemic corticosteroids by 36% [6]. A subgroup analysis revealed that vitamin D supplementation reduced the frequency of asthma exacerbations requiring systemic corticosteroids in individuals with an initial 25-hydroxyvitamin D level below 25 nmol/L. However, no such effect was observed in individuals with a baseline 25-hydroxyvitamin D level of 25 nmol/L or higher [6]. Wang et al. demonstrated that forced expiratory volume (FEV1) improved in patients with vitamin D deficiency and air limitation (FEV1 < 80%). The administration of vitamin D supplements resulted in a 27% reduction in asthma aggravations, particularly among patients who had vitamin D deficiency [27]. Furthermore, vitamin D had a favorable effect on pulmonary function in patients with air restriction and vitamin D deficiency. As a viable treatment approach, vitamin D supplementation is a low-cost, low-risk method of treating and controlling asthma [27]. A post-hoc analysis of an RCT by Camargo et al. showed that, although monthly high-dose vitamin D supplementation had no overall effect on asthma attacks, there was evidence of a possible benefit for people with severe vitamin D deficiency [28]. Although additional research is required to confirm this finding, Riverin et al. reported moderate evidence from one trial that monthly doses of 60,000 IU of vitamin D may help in avoiding ED visits [29]. Solidoro et al. said that vitamin D deficiency in asthmatic patients encourages exacerbations and interferes with asthma control. The discovery that long-term vitamin supplementation, in addition to conventional asthma therapy, lowers exacerbations in individuals with vitamin D insufficiency points to the possibility that monitoring vitamin levels and bringing them back to normal levels could be a straightforward method for enhancing asthma management [30]. The objective of vitamin D administration should be to elevate and maintain levels of 25-hydroxyvitamin D above 30 ng/mL throughout the entire year. To raise the blood level of 25-hydroxyvitamin D above 30 ng/mL in patients with deficiency, a daily dose of 1500-2000 IU should be adequate [30]. With a more pronounced risk reduction of 46% in women with a 25-hydroxyvitamin D level of 30 ng/ml at trial entry, Wolsk et al. combined analysis of RCTs demonstrate a significant and clinically important 26% protective effect of vitamin D supplementation during pregnancy on the risk of asthma or recurrent wheeze in the offspring [31]. Therefore, prenatal care programs that aim to increase pregnant women's vitamin D levels should be taken into consideration [31].

Limitations

The study has some limitations, including most of the positive findings that showed protective effects came from meta-analyses. All the RCTs reported showed no benefit to supplement with vitamin D, which indicates a scarcity and need for high-level evidence such as RCTs. All of the studies identified were based on the limited number of clinical trials that were available. All of the studies showed variations in sample size and variable measurement. Not all of the studies examined had the same variables and secondary outcomes. This review included only papers written in English; thus, information from papers written in languages other than English was excluded.

## Conclusions

Over the past few decades, there has been much debate about the effectiveness of vitamin D supplementation for asthmatics. Extensive research conducted in both laboratory settings and living organisms have demonstrated the robust immunomodulatory properties of vitamin D. Both children and adults have provided substantial evidence demonstrating the favorable advantages of vitamin D supplementation in individuals with asthma who exhibit insufficient levels of vitamin D. The role of vitamin D in asthmatics with adequate vitamin D levels is still up for debate. Despite this, we advise doctors to have a low threshold for prescribing vitamin D to vitamin D-deficient asthma patients until adequate levels are reached due to the cost-effectiveness of vitamin D; 1500-2000 IU per day should be sufficient. Therefore, there is a need for focused, high-quality clinical trials with adequate sample sizes in the years to come. The optimum vitamin D level for different age groups, genders, ethnicities, and asthma phenotypes should be considered when planning trials. Trials will undoubtedly provide more detailed information on whether vitamin D supplementation should be abandoned as yet another ineffective asthma treatment approach or used as a mainstay therapy for airway remodeling in conjunction with other available therapeutic options.
